# Metabolically healthy obese women have longer telomere length than obese women with metabolic syndrome

**DOI:** 10.1371/journal.pone.0174945

**Published:** 2017-04-06

**Authors:** Andrea E. Iglesias Molli, Julieta Panero, Patricia C. Dos Santos, Claudio D. González, Jorge Vilariño, Marta Sereday, Gloria E. Cerrone, Irma Slavutsky, Gustavo D. Frechtel

**Affiliations:** 1 CONICET-Universidad de Buenos Aires. Instituto de Inmunología, Genética y Metabolismo (INIGEM). Laboratorio de Diabetes y Metabolismo. Buenos Aires, Argentina; 2 Academia Nacional de Medicina. CONICET. Instituto de Medicina experimental (IMEX). Laboratorio de Genética de Neoplasias Linfoides. Buenos Aires, Argentina; 3 Universidad de Buenos Aires. Facultad de Medicina. Departamento de Farmacología. Cátedra II. Buenos Aires, Argentina; 4 Hospital FLENI. Departamento de Cardiología. Buenos Aires, Argentina; 5 Hospital Fiorito. Servicio de Endocrinología. Buenos Aires, Argentina; 6 Universidad de Buenos Aires. Facultad de Farmacia y Bioquímica. Departamento de Microbiología, Inmunología y Biotecnología. Cátedra de Genética. Buenos Aires, Argentina; CHA University, REPUBLIC OF KOREA

## Abstract

**Introduction:**

Obesity is the principal component in the Metabolic Syndrome (MetS) that determines the progression of metabolic complications. Metabolically healthy obese (MHO) individuals seem to be protected against those complications. Telomere length (TL) as a novel marker of cellular aging had a complex relationship to the MetS. The principal aim of this study was to investigate the TL in MHO, and to study the association between TL and the worsening of the metabolic condition.

**Material and methods:**

We have determined the absolute TL (aTL) in 400 women (mean age of 46.76 ± 15.47 years; range: 18–86 years), grouped according to the metabolic condition in three groups: metabolically healthy non-obese women (MHNO), MHO and obese women with MetS (MSO); and grouped according to the number of components of MetS.

**Results:**

We found that MHO displays significantly higher aTL than MSO (p = 0.033; r = -4.63; 95% CI r = -8.89 / -0.37), but did not differ from MHNO. A decrease in aTL with the progressive increase in the number of MetS components was also observed (p < 0.001; r = -2.06; 95% CI r = -3.13 / -0.99). In this way, our results indicate that aTL is influenced by the presence of MetS, but it is not affected by the presence of obesity.

**Discussion:**

We found that shorter aTL is not associated with MHO, but is related to MetS and with the increased number of metabolic abnormalities.

## Introduction

The Metabolic Syndrome (MetS) is a cluster of cardiovascular and metabolic risk factors including central obesity, hypertension, hyperglycemia and dyslipidemia. These well-known risk factors co-segregate in an individual more often than might be expected by chance, and predispose to type 2 diabetes mellitus and to cardiovascular disease (CVD). The etiology of MetS is not yet well understood, but predisposing factors include aging, inflammation, obesity, sedentary, lifestyle and genetics [1]. The insulin resistance is likely to be a significant link between the components of MetS. Increased oxidative stress and inflammation have emerged as playing a central role in MetS and may be a unifying factor in the progression of this disease. Central obesity is the principal component of the MetS that determinates the progression of those metabolic complications. However, a subgroup of obese individuals seems to be protected against obesity-related metabolic complications [2]. These individuals are described as metabolically healthy obese (MHO), or as having uncomplicated obesity [3]. It is a new concept in which an individual may exhibit an obese phenotype without the presence of MetS. The existence of MHO phenotype was first proposed by Sims in 2001; it is considered that a proportion between 15% and 20% of obese individuals may be free of metabolic complications during an unknown period of time [4]. The subjects with MHO do not exhibit at baseline an increased mortality, or an increased risk of CVD or type 2 diabetes compared to normal-weight controls [5].

Telomere length (TL), generally assessed in leukocytes, is a novel marker of cellular aging and has been associated with increased risks of morbidity and mortality [6,7]. Telomeres are nucleoprotein complexes located at the ends of eukaryotic chromosomes, composed of DNA tandem repeats (TTAGGG in mammals) and associated proteins. They play an essential role in preserving chromosomal integrity and stability, constituting a critical factor in cell survival [8]. Telomeric DNA erodes with each cell division by incomplete replication of the lagging strand during DNA synthesis, known as the “end-replication problem”. Through this process, each telomeric end shortens by approximately 20–60 base pairs (bp)/year in peripheral blood lymphocytes [9–11]. In vitro studies have found that when telomeres become critically short, cells stop dividing and promote replicative senescence, contributing to ageing and subsequent somatic cells death [12]. Cellular damage due to increased oxidative stress [13] can further accelerate the process of TL reduction. Therefore, TL can be considered as a biomarker of aging, where shorter TL would indicate increased biological age. Indeed, metabolic disorders such as MetS, which is aging related, display functional decline in tissues such as the heart, liver and pancreas that are well-recognized features in aged individuals [14,15]. Some studies have shown significant associations between shorter TL and central adiposity [16]. In this way, the TL was shorter according to the worsening of the metabolic condition in a representative sample of females [17]. It is known that there is a complex relationship between TL with obesity and the MetS components, but we do not know the impact of the TL in particular groups of obesity such as MHO. In this context, the principal aim of this study was to investigate the absolute TL (aTL) in MHO individuals, who were compared with a control group composed of non-obese individuals without MetS and with a cohort of patients with obesity and MetS.

## Material and methods

### Population

From the general population of Venado Tuerto, Province of Santa Fe, 1098 adult individuals of both sexes were selected randomly in 2011 following a stratified multistage sampling design, in accordance to the latest population census performed in 2010. Of the total population, 637 individuals agreed to participate in the study, and we carried out this work only in women (n = 400) taking into account gender differences in aTL [18,19]. The study was approved by the Ethics Committee of Venado Tuerto Hospital and all participants gave their written informed consent. The prevalence of chronic metabolic diseases and the study of biochemical markers associated with CVD in the population are already published [20]. MHO group was defined as individuals with obesity but without MetS [21]. For the diagnosis of obesity, body mass index (BMI) ≥ 30 kg / m^2^ was considered. The diagnosis of MetS was performed according to the ATPIII criteria [22], as individuals with three or more of the following components for women: waist circumference ≥ 88 cm; fasting plasma glucose (FPG) ≥ 100 mg dl-1 or individual treated for type 2 diabetes; systolic blood pressure (SBP) ≥ 130 mmHg / diastolic blood pressure (DBP) ≥ 85 mmHg or individual treated for hypertension; high-density lipoprotein cholesterol (HDL-C) < 50 mg dl-1 and triglycerides (TG) ≥ 150 mg dl-1 or individual treated for dyslipidemia. For aTL study, MHO women were compared with two other groups: obese women with MetS (MSO), a group with worse metabolic condition, consisting of individuals with both MetS and obesity; and metabolically healthy non-obese women (MHNO), a group with better metabolic condition, composed of individuals without obesity and MetS. In addition, we regrouped our population based on the number of components of MetS, into groups of individuals without any component of MetS (0), with one component (1), two components (2) and MetS with three or more components.

As TL was reported to be associated with the level of physical activity [23] and the lifetime accumulated exposure to tobacco [24], all subjects completed a questionnaire detailing their self-reported physical activity and cigarette smoking habits. It was considered the physical activity level during leisure time and at work, and it was classified into 3 categories, where 1 indicates inactive; 2, light to moderate activity; and 3, heavy activity. To measure the exposure to tobacco, pack-years smoked were calculated as the number of packs (20 cigarettes) smoked per day multiplied by years of smoking.

Anthropometric measurements (height, weight and waist circumference), and SBP and DBP were determined by a standardized protocol. BMI was calculated as weight (kg) / [height (m)]^2^. After a 12 h overnight fast, venous blood samples (20 mL) were drawn in every individual. One aliquot of blood was frozen at -20°C and reserved for DNA extraction. The remaining aliquot was centrifuged to obtain serum and analyzed immediately. FPG was determined by a glucose-oxidase method (GLU Glucose GOD-PAP, Roche Diagnostics, Mannheim, Germany) in a Hitachi 727 auto-analyzer. Fasting serum insulin (FSI) was measured by electrochemiluminescence immunoassay with a commercial kit (Insulin, Roche Diagnostics, Mannheim, Germany) in a Cobas e411 (Roche Diagnostics, Mannheim, Germany), with a lower detection limit of 2 μU/mL. As an indicator of insulinresistance we used the homoeostasis model assessment (HOMA), calculated as FPG (mM) * FSI (mIU/L) / 22.5. TG and HDL-C were determined in serum using standardized procedures by enzymatic methods using commercial kits (TG Triglycerides GPO-PAP, and Phosphotungstate Precipitant and CHOL Cholesterol CHOD-PAP, Roche Diagnostics, Mannheim, Germany) by using a Hitachi 727 auto-analyzer. Intra-CV (coefficient of variation) for TG and HDL-C were 1.3% and 1.1%, respectively. Inter-CVs were 2.4% and 1.5%, respectively. Serum high-sensitivity C reactive protein (hs-CRP) levels were measured using particle enhanced immunonephelometry assay (Cardio Phase® hsCRP, Siemens) on a Siemens BN Prospect Nephelometer (Siemens Healthcare Diagnostics, Deerfield, IL, USA). We considered that hs-CRP values >10 mg/l were likely to represent an acute inflammatory response in line with previous studies [25].

### Measurement of absolute TL

We carried out the aTL measurement by qPCR as previously described [26], in a LightCycler system (Roche Diagnostics, Mannheim, Germany), which was previously validated by our working group [27]. Genomic DNA was extracted from peripheral blood leucocytes. For each DNA sample, two consecutive reactions were performed: the first one to amplify a fragment of 75 bp of the single copy gene RPLPO (ribosomal protein, large, PO), and the second one for the telomeric sequence. Primers sequences for RPLPO PCR were 5’-CAGCAAGTGGGAAGGTGTAATCC-3' (forward), and 5’-CCCATTCTATCATCAACGGGTACAA-3' (reverse); and for the telomere PCR were 5’-CGGTTTGTTTGGGTTTGGGTTTGGGTTTGGGTTTGGGTT-3' (forward), and 5’-GGCTTGCCTTACCCTTACCCTTACCCTTACCCAATCCCT-3' (reverse). Both PCRs were performed in a final volume of 20 μl containing 20 ng DNA, 1X SYBR Green Master Mix (Roche Diagnostics, Mannheim, Germany) and 250 nM RPLPO primers or 100 nM telomere primers. The PCR conditions were: 10 min at 95°C, followed by 45 cycles of 15 sec at 95°C, 1 min at 60°C. The melting curve was performed with 1 cycle of 20 sec at 95°C, 15 sec 50°C, and 98°C with a temperature ramp of 0.1°C/sec. Each sample was analyzed in duplicate, and all measurements included the determination of the standards and a no-template negative control, in which the DNA was substituted by water. We run a standard curve in each PCR, constructed using ten-fold serial dilutions of synthesized oligonucleotides. For the RPLP0 PCR we used the RPLP0 PCR product as standard, and the export values from the standard curves were diploid genome copies/reaction. The standard for telomere PCR is an oligomer containing only the telomeric DNA tandem repeats (TTAGGG repeated 14 times), and the export values from the standard curves were kbp of telomeric sequences/reaction. The kbp/reaction value was divided by the diploid genome copy number/reaction to calculate the aTL in kbp per human diploid genome, and then divided by the 92 telomeres present in a human genome to inform the aTL in kbp per telomere. To evaluate the reliability of the assay we calculated the intra and inter-CV, and they were sufficiently low for the RPLPO PCR (0.6% and 3.9%, respectively) and for the telomere PCR (1.6% and 10.4%, respectively).

### Statistical evaluation

Statistical analyses were performed using SPSS version 20.0 (IBM Corp., Armonk, NY, USA). We used one way-ANOVA and Bonferroni *post-hoc* test to compare biochemical, clinical and anthropometric characteristics between groups. In agreement with the Central Limit Theorem, due to the number of included patients, these variables were treated as normally distributed. Linear regression was performed to evaluate the relationship between aTL and age for the general population. The analysis of aTL in the groups MHNO, MHO and MSO, was performed by multiple linear regression considering the groups as ordinal variable, since each group shows a worse metabolic condition than the previous one; and the study of dummy variables to compare the aTL between every pair of groups. In this analysis, the age-squared, pack-years smoked and physical activity were used as covariates, as they were associated with aTL. The same analysis was performed to evaluate the relationship between the aTL and the number of components of MetS. A p value below 0.05 was considered as statistically significant.

## Results

The overall population consisted of 400 women with a mean age of 46.76 years (SD = 15.47; range: 18–86 years). Taking into account biochemical, clinical and anthropometric measurements, we observed that 49.0% of the cohort (n = 196) were MHNO, 10.5% (n = 42) MHO and 25.5% (n = 102) MSO. The remaining 15.0% of the cohort (n = 60) did not display the characteristics of any group, thus they were not included for this part of the study. All the variables analyzed showed significant differences among the three groups (Table 1). The *post-hoc* analysis between MHNO and MHO, among which the only difference is the presence of obesity, showed differences in the parameters related to body weight: BMI and waist circumference. They also showed differences in SBP, DBP and hs-CRP. The other components of MetS (FPG, TG and HDL-C), FSI and HOMA showed no differences between MHNO and MHO. On the other hand, when we compared MHO with MSO women, similar values in BMI and hs-CRP were observed, but they differ in every component of MetS (waist circumference, FPG, HDL-C, TG, SBP and DBP), as expected considering that both groups are of obese individuals and the difference between them are the presence of MetS. The MSO group also showed significantly higher values in the HOMA and FSI compared to MHO group. The comparison between MHNO and MSO groups showed differences in all the biochemical-clinical parameters analyzed. We also found significant differences in age when comparing the three groups, where the MSO group presented significantly higher age than the other two groups.

**Table 1:** Characteristics of the population grouped according to the metabolic condition.

	Groups			Statistical comparison	Test *post-hoc*								
	MHNO	MHO	MSO		MHNO vs MHO			MHO vs MSO			MHNO vs MSO		
	M ± SD	M ± SD	M ± SD	P	MD	95% CI MD	p	MD	95% CI MD	p	MD	95% CI MD	p
n	196	42	102										
Age (years)	42 ± 15	44 ± 15	51 ± 14	< 0.001	-1.94	-7.91/4.02	NS	-7.04	-13.47/-0.6	0.027	-8.98	-13.26/-4.69	< 0.001
BMI (kg m^-2^)	24.52 ± 2.95	35.33 ± 6.65	36.94 ± 6.17	< 0.001	-10.82	-12.73/-8.91	< 0.001	-1.61	-3.67/0.45	NS	-12.43	-13.80/-11.06	< 0.001
WC (cm)	86 ± 9	106 ± 10	112 ± 11	< 0.001	-19.53	-23.61/-15.45	< 0.001	-5.97	-10.37/-1.56	0.004	-25.49	-28.43/-22.56	< 0.001
FPG (mg dl^-1^)	89 ± 10	90 ± 8	115 ± 49	< 0.001	-1.37	-12.86/10.12	NS	-24.55	-36.94/-12.16	< 0.001	-25.92	-34.17/-17.67	< 0.001
FSI (mcU ml-1)	9.95 ± 7.44	12.91 ± 7.99	19.01 ± 15.99	< 0.001	-2.96	-7.39/1.47	NS	-6.10	-10.87/-1.33	0.007	-9.06	-12.24/-5.88	< 0.001
HOMA	2.23 ± 1.79	2.88 ± 1.79	5.53 ± 6.2	< 0.001	-0.65	-2.17/0.87	NS	-2.65	-4.29/-1.01	< 0.001	-3.31	-4.40/-2.21	< 0.001
HDL-C (mg dl^-1^)	57 ± 13	55 ± 12	47 ± 12	< 0.001	2.37	-2.95/7.69	NS	7.97	2.23/13.71	0.003	10.34	6.52/14.16	< 0.001
TG (mg dl^-1^)	97 ± 42	92 ± 36	166 ± 97	< 0.001	4.59	-21.44/30.62	NS	-73.99	-102.05/-45.93	< 0.001	-69.40	-88.09/-50.71	< 0.001
SBP (mmHg)	111 ± 19	121 ± 20	137 ± 21	< 0.001	-9.64	-17.64/-1.65	0.012	-15.45	-24.07/-6.83	< 0.001	-25.09	-30.83/-19.35	< 0.001
DBP (mmHg)	69 ± 12	75 ± 14	83 ± 11	< 0.001	-6.11	-10.92/-1.29	0.007	-8.13	-13.31/-2.94	0.001	-14.23	-17.69/-10.77	< 0.001
hs-CRP (mg l^-1^)	2.01 ± 2.14	3.18 ± 2.60	3.89 ± 2.57	< 0.001	-1.17	-2.23/-0.12	0.024	-0.70	-1.85/0.44	NS	-1.88	-2.62/-1.14	< 0.001

Statistical evaluation: One way-ANOVA and Bonferroni *post-hoc* test.

MHNO: Metabolically Healthy Non-obese individuals; MHO: Metabolically Healthy Obese individuals; MSO: Obese individual with Metabolic Syndrome; M: mean; SD: standard deviation; MD: means difference; 95% CI MD: 95% confidence interval of the means difference; BMI: Body mass index; WC: Waist circumference; FPG: fasting plasma glucose; FSI: Fasting serum insulin; HOMA: Homoeostasis Model Assessment; HDL-C: high-density lipoprotein cholesterol; TG: triglycerides; SBP: systolic blood pressure; DBP: diastolic blood pressure; hs-CRP: High-Sensitive C reactive protein; NS: not significant.

**Fig 1:**
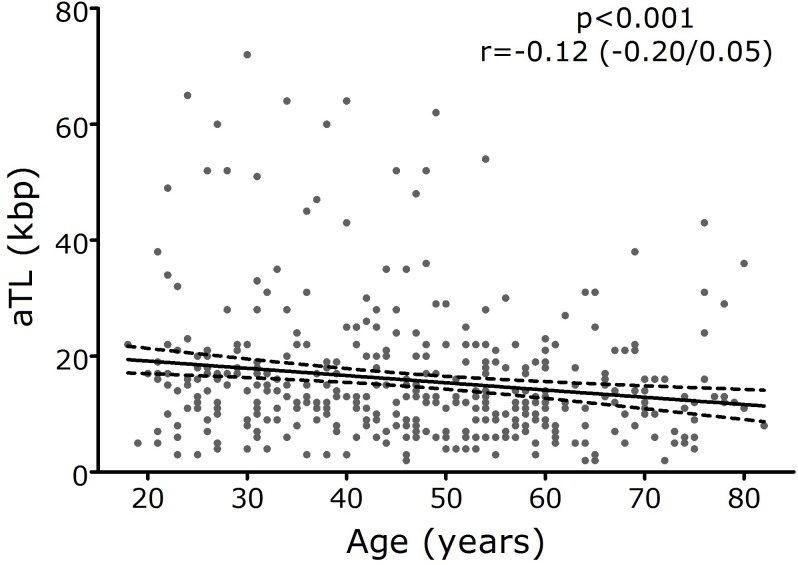
Telomere length as a function of age for the general population. Statistical evaluation: Linear regression. Mean (─) and standard deviation (-). aTL: Absolute telomere length.

The analysis of aTL showed a median value of 12.95 kbp (range: 1.57–72.12 kbp) for the general population. The relationship between aTL and age was evaluated and a significant shortening of aTL with age progression was observed in the general population (p = 0.001; r = -0.12; 95% CI r = -0.20 / -0.05) (Fig 1).

**Fig 2:**
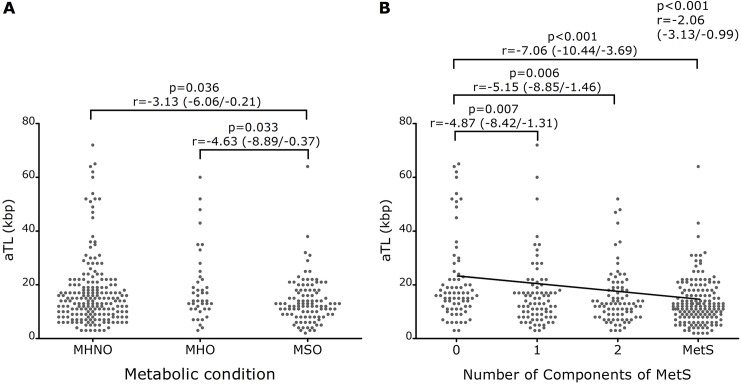
**Telomere length according to the metabolic condition (A) and according to the number of components of metabolic syndrome (B).** Statistical evaluation: Multiple linear regression and the study of dummy variables. Covariates: age, pack-years smoked and physical activity. aTL: Absolute telomere length; MHNO: Metabolically Healthy Non-obese individuals; MHO: Metabolically Healthy Obese individuals; MSO: Obese individual with Metabolic Syndrome; MetS: Metabolic Syndrome (individual with three or more components).

The median aTL was 14.26 kpb (range: 2.66–72.12 kpb) for MHNO, 14.11 kpb (range: 2.83–60.38 kpb) for MHO, and 12.51 kpb (range: 1.57–64.39 kpb) for MSO individuals. Although the median aTL tend to decrease as the metabolic condition worsens, it did not archive statistical significance (p = 0.054; r = -1.43; 95% CI r = -2.88 / 0.02). The comparison of aTL among groups showed that MSO had significantly shorter aTL than MHNO (p = 0.036; r = -3.13; 95% CI r = -6.06 / -0.21), and MHO (p = 0.033; r = -4.63; 95% CI r = -8.89 / -0.37), but we found no differences in aTL between MHNO and MHO (p = 0.456, r = -1.50; 95% CI r = -5.44 / 2.45) (Fig 2A), indicating that aTL was influenced by the presence of MetS, but it was not affected by the presence of obesity. All these results were performed with age-squared, tobacco consumption and physical activity corrections. Thus, in order to evaluate the relationship between aTL and the number of MetS components, we regrouped the population according to the number of components of the MetS.

From the total of 400 women of the general population, 18.5% (n = 74) had no components of MetS (group 0), 20.0% (n = 80) showed one component (group 1), 21.0% (n = 84) had two components (group 2) and 40.5% (n = 162) showed three or more components (group MetS). All biochemical-clinical characteristics analyzed showed significant differences among the four groups (Table 2). In the *post-hoc* comparison the group with MetS differed from all other groups without MetS (0, 1 and 2) in all variables analyzed.

**Table 2:** Characteristics of the population grouped according to the Metabolic Syndrome number of components.

**A**	**MetS number of components**							**Statistical comparison**	
	**0**		**1**		**2**	**MetS**			
	M ± SD		M ± SD		M ± SD	M ± SD		P	
n	74		80		84	162			
Age (years)	35 ± 10		39 ± 15		51 ± 14	53 ± 14		< 0.001	
BMI (kg m^-2^)	24.23 ± 3.06		26.31 ± 5.34		28.48 ± 6.86	33.12 ± 7.14		< 0.001	
WC (cm)	84 ± 10		89 ± 11		95 ± 13	105 ± 14		< 0.001	
FPG (mg dl^-1^)	86 ± 8		89 ± 8		92 ± 11	112 ± 44		< 0.001	
FSI (mcU ml^-1^)	10.14 ± 7.93		9.11 ± 5.88		12.07 ± 8.54	16.38 ± 13.96		< 0.001	
HOMA	2.18 ± 1.81		2.02 ± 1.34		2.80 ± 2.08	4.68 ± 5.25		< 0.001	
HDL-C (mg dl^-1^)	64 ± 11		52 ± 12		54 ± 14	47 ± 13		< 0.001	
TG (mg dl^-1^)	78 ± 28		89 ± 31		117 ± 50	170 ± 97		< 0.001	
SBP (mmHg)	103 ± 12		113 ± 20		122 ± 19	135 ± 21		< 0.001	
DBP (mmHg)	65 ± 8		70 ± 12		76 ± 13	81 ± 12		< 0.001	
hs-CRP (mg l^-1^)	1.70 ± 1.69		2.43 ± 2.49		2.43 ± 2.44	3.73 ± 2.48		< 0.001	
**B**	**Test *post-hoc***								
	**0 vs 1**			**0 vs 2**			**1 vs 2**		
	MD	95% CI MD	p	MD	95% CI MD	p	MD	95% CI MD	p
Age (years)	-4.02	-9.84/1.81	NS	-15.43	-21.19/-9.67	< 0.001	-11.42	-17.06/-5.77	< 0.001
BMI (kg m^-2^)	-2.08	-4.72/0.55	NS	-4.25	-6.85/-1.64	< 0.001	-2.17	-4.72/0.38	NS
WC (cm)	-4.35	-9.57/0.87	NS	-10.68	-15.84/-5.52	< 0.001	-6.33	-11.39/-1.28	0.006
FPG (mg dl^-1^)	-3.43	-15.69/8.83	NS	-6.42	-18.53/5.7	NS	-2.98	-14.86/8.89	NS
FSI (mcU ml^-1^)	1.03	-3.56/5.61	NS	-1.94	-6.47/2.6	NS	-2.96	-7.37/1.45	NS
HOMA	0.16	-1.4/1.71	NS	-0.62	-2.16/0.92	NS	-0.78	-2.27/0.72	NS
HDL-C (mg dl^-1^)	11.50	6.16/16.84	< 0.001	9.46	4.18/14.74	< 0.001	-2.04	-7.2/3.11	NS
TG (mg dl^-1^)	-10.76	-40.1/18.58	NS	-38.81	-67.81/-9.8	0.003	-28.05	-56.47/0.37	NS
SBP (mmHg)	-9.71	-17.79/-1.63	0.009	-19.27	-27.25/-11.28	< 0.001	-9.56	-17.38/-1.73	0.008
DBP (mmHg)	-5.13	-10.04/-0.21	0.036	-10.84	-15.69/-5.98	< 0.001	-5.71	-10.47/-0.96	0.009
hs-CRP (mg l^-1^)	-0.73	-1.78/0.33	NS	-0.73	-1.77/0.3	NS	0.00	-1.05/1.04	NS
**C**	**Test *post-hoc***								
	**0 vs MetS**			**1 vs MetS**			**2 vs MetS**		
	MD	95% CI MD	p	MD	95% CI MD	p	MD	95% CI MD	p
Age (years)	-17.89	-22.96/-12.82	< 0.001	-13.87	-18.81/-8.93	< 0.001	-2.45	-7.31/2.41	NS
BMI (kg m^-2^)	-8.90	-11.19/-6.6	< 0.001	-6.82	-9.05/-4.58	< 0.001	-4.65	-6.84/-2.45	< 0.001
WC (cm)	-20.51	-25.06/-15.97	< 0.001	-16.16	-20.59/-11.73	< 0.001	-9.83	-14.18/-5.47	< 0.001
FPG (mg dl^-1^)	-25.83	-36.51/-15.16	< 0.001	-22.40	-32.8/-12	< 0.001	-19.42	-29.65/-9.19	< 0.001
FSI (mcU ml^-1^)	-6.25	-10.25/-2.25	< 0.001	-7.28	-11.13/-3.42	< 0.001	-4.31	-8.11/-0.52	0.017
HOMA	-2.51	-3.87/-1.15	< 0.001	-2.66	-3.98/-1.35	< 0.001	-1.89	-3.18/-0.6	0.001
HDL-C (mg dl^-1^)	16.55	11.90/21.21	< 0.001	5.05	0.54/9.56	0.019	7.10	2.65/11.53	< 0.001
TG (mg dl^-1^)	-91.59	-117.14/-66.04	< 0.001	-80.84	-105.72/-55.95	< 0.001	-52.79	-77.27/-28.3	< 0.001
SBP (mmHg)	-31.64	-38.66/-24.61	< 0.001	-21.93	-28.77/-15.08	< 0.001	-12.37	-19.1/-5.64	< 0.001
DBP (mmHg)	-16.50	-20.77/-12.23	< 0.001	-11.37	-15.53/-7.21	< 0.001	-5.66	-9.75/-1.57	0.002
hs-CRP (mg l^-1^)	-2.03	-2.94/-1.12	< 0.001	-1.30	-2.22/-0.38	0.001	-1.30	-2.19/-0.41	0.001

Statistical evaluation: One way-ANOVA (A) and Bonferroni *post-hoc* test (B-C).

MetS: Metabolic Syndrome; M: mean; SD: standard deviation; MD: means difference; 95% CI MD: 95% confidence interval of the means difference; BMI: Body mass index; WC: Waist circumference; FPG: fasting plasma glucose; FSI: Fasting serum insulin; HOMA: Homoeostasis Model Assessment; HDL-C: high-density lipoprotein cholesterol; TG: triglycerides; SBP: systolic blood pressure; DBP: diastolic blood pressure; hs-CRP: High-Sensitive C reactive protein; NS: not significant.

The median aTL was 16.35 kpb (range: 2.86–64.99 kpb) for individuals with no components of MetS, 13.24 kpb (range: 2.66–72.12 kpb) for individuals with one component, 12.75 kpb (range: 2.66–51.57 kpb) for individuals with two components, and 11.91 kpb (range: 1.57–64.39 kpb) for individuals with MetS. It can be seen that the median aTL is shorter with the presence of MetS abnormalities, and that it becomes shorter with the increase in the number of components of the MetS. In addition, we observed statistically significant association between aTL and the number of components of MetS with age-square, tobacco consumption and physical activity corrections (p < 0.001; r = -2.06; 95% CI r = -3.13 / -0.99) (Fig 2B). The comparison between groups showed that individual with no components of MetS (group 0) had aTL significantly longer than the other groups (0 vs 1: p = 0.007, r = -4.87, 95% CI r = -8.42 / -1.31; 0 vs 2: p = 0.006, r = -5.15, 95% CI r = -8.85 / -1.46; 0 vs MetS: p < 0.001, r = -7.06, 95% CI r = -10.44 / -3.69), with no differences between groups 1, 2 and MetS (1 vs MetS: p = 0.173, r = -2.19, 95% CI r = -5.35 / 0.97; 2 vs MetS: p = 0.208, r = -1.91, 95% CI r = -4.88 / 1.06; 1 vs 2: p = 0.874, r = -0.29, 95% CI r = -3.82 / 3.25), reinforcing the idea that the presence of at least one of the MetS components is associated with a shorter aTL.

## Discussion

This is the first description about aTL in a population of MHO patients compared to MSO and MHNO individuals. To our knowledge, this is the first report showing that MHO group had no differences in aTL with respect to the MHNO group, but it shows significant differences with respect to the MSO group.

MHO group would be defined as a subgroup of obese patients with an intermediate phenotype between MHNO and MSO individuals. In this way, when we compared MHO and MSO groups we found that both had similar BMI, but they differ in every component of MetS. The difference in waist circumference demonstrates the importance of central obesity in the development of MetS. Actually, it is accepted that visceral and ectopic fat content has been associated with a decrease in insulin sensitivity, which could play an important role in the development of metabolic complications and increase risk of CVD. In that sense, we observed that although MHO individuals have large quantities of fat mass, they might present remarkably normal to high levels of insulin sensitivity. This could be due to MHO individuals which show 49% less visceral adipose tissue (as measured from computed tomography) than at risk subjects with the MetS [28]. On the other hand, when we compared MHNO and MHO they showed differences in BMI, waist circumference and blood pressure, but not in the other components of MetS. We could see that MHO had similar CRP than MSO, as recently found in Brazilian population [29]. Also, when we evaluated the insulin resistance, we found that MHO had significantly lower HOMA index compared to MSO patients, and that MHO and MHNO did not differ with respect to insulin sensibility. Similar results have been published in metabolically healthy peripherally obese [30].

When MHO was followed up during longitudinal studies in order to investigate the cardiovascular risk and mortality, results showed that MHO group is a clinical entity in between healthy non-obese individuals and MetS individuals. A 7-years prospective study found that MHO subjects did not have an increased risk for CVD or all-cause mortality compared with healthy non-obese individuals [31]. However, another study with a follow-up period of 7.4 years revealed that obese participants without MetS had higher risk of major cardiovascular events compared with metabolically healthy non-obese subjects but less than unhealthy metabolic non-obese individuals [32]. A recent meta-analysis that evaluated participants for all-cause mortality and/or CVD showed an increased risk for CVD in MHO individuals compared to metabolically healthy normal weight individuals (OR [Odds ratio] = 1.24, 95% CI = 1.02–1.55), but fewer than obese unhealthy individuals (OR = 2.65; 95% CI = 2.18–3.12) [33]. Furthermore, the patients with coronary artery disease who presented MetS had shorter TL than patients without MetS [34].

Our molecular analysis showed that aTL in MHO group is similar to that observed in MHNO and larger than in MSO individuals, supporting the finding that those patients have a better metabolic behavior than MetS individuals.

Another finding of our work was that the aTL is dependent upon the presence of metabolic abnormalities. Thus, we could associate shorter aTL with the raise in the number of MetS components, supporting a relationship between telomere shortening and the metabolic severity. In agreement with our results, a recent report [35] found that shorter TL was related with a higher number of MetS abnormalities representing a more severe metabolic profile. Although this association, found at baseline of the study, was reduced over two to six years follow up, shorter baseline TL was still significantly associated with unfavorable scores of most MetS components. In addition, Njajou et al [36] published similar findings, where a seven years follow up was associated with smaller increases in BMI and body fat in those individuals who presented longer TL. Rehkopf et al [37] in a large nationally representative U.S. sample did not find an association between MetS and shorter TL. They did not report the prevalence of MetS in their population and not stratify the individuals in accordance to the variables of MetS, as we performed in our study. Although we found shorter aTL in MSO and an association with the number of MetS components, like Rehkopf we could not associate MetS with shorter aTL. In this sense, we had not found association between TL and the individual components of MetS (data not shown). Anyway, we must consider genetics, ethnics, demographics, feeding and behaviors differences between both populations that may interfere the relationship between TL and MetS.

Several issues may influence the TL; some of them are not modifiable such as age, inheritance, gender and ethnicity, among the most important ones. On the other hand, there are modifiable clinical variables that may determine the TL, such as high FPG, increase BMI and waist circumference, high TG, reduced HDL-C, smoking, physical activity, and diet. In this respect, different studies showed the relationship between TL and metabolic abnormalities [38–40]. In our study, we could associate the number of metabolic alterations with aTL. In this respect, molecular mechanism such as inflammation and oxidative damage could lead to short telomeres as a consequence of a worsened metabolic state as we demonstrated in this work [41]. Interestingly, the increasing in abdominal adiposity was found to be accompanied by shorter TL, in the same way we found that MHO had a waist circumference lowest than MSO [42]. In this regards, recently an Editorial has been published about MHO which includes definition, metabolic outcomes, molecular genetics findings and therapeutic interventions [43]. This paper claims the need about longitudinal trials in individuals who are MHO at baseline and determine the development of the typical metabolic complications of obesity and the molecular genetics mechanisms contributing toward its development. Beyond whether MHO is or not a clinical entity, we could associate the shorter aTL to the increased presence of metabolic disorders, emphasizing the importance of its clinical management to prevent cardiovascular disease.

Martin-Ruiz et al [44] published that the variation of the TL and the amount of methodological differences between laboratories is large and it has been demonstrated a huge inter-laboratory variation even for relative TL following internal normalization. In general, it must be considered that the same reference range for TL cannot be applied by all laboratories. So, TL measurement of an individual or a group of individuals could only be useful as a risk indicator if reference values were measured by the same laboratory using the same protocols.

In conclusion, we are showing for the first time that aTL in MHO women is similar to that in MHNO women, and longer than that observed in MSO women, suggesting its importance in the interpretation of physiopathology and treatment of this subtype of obesity [45]. Our work has some limitations: (i) it was carried out only in women, (ii) we studied the aLT average of all white blood cells in peripheral circulation without controlling for cell type, (iii) oxidative stress molecular markers were not determined. Nevertheless, our report indicates that: (i) some metabolic conditions such as MHO, is not associated with shorter aTL; and (ii) shorter aTL is related to MetS and with the increased number of metabolic abnormalities, representing a more severe metabolic profile and finally that the shortening of the aTL throughout life is less pronounced in individuals with normal weight and without metabolic abnormalities. Although some findings are consistent with a role for TL in the etiology of CVD and MetS, is unclear the exact relationship between them [46]. We thought that the TL shortens as a consequence of multiple types of biochemical stressors and may represent an index of several known risk factors. In this way, our study may contribute to this matter.

## References

[1] Gallagher EJ, LeRoith D, Karnieli E. The metabolic syndrome-from insulin resistance to obesity and diabetes. Endocrinol Metab Clin North Am 2008;37:559–579. doi: 10.1016/j.ecl.2008.05.002 18775352 10.1016/j.ecl.2008.05.002

[2] Karelis AD, St-Pierre DH, Conus F, Rabasa-Lhoret R, Poehlman ET. Metabolic and body composition factors in subgroups of obesity: what do we know? J Clin Endocrinol Metab 2004;89:2569–2575. doi: 10.1210/jc.2004-0165 15181025 10.1210/jc.2004-0165

[3] Iacobellis G, Ribaudo MC, Zappaterreno A, Iannucci CV, Leonetti F. Prevalence of uncomplicated obesity in an Italian obese population. Obes Res 2005;13:1116–1122. doi: 10.1038/oby.2005.130 15976155 10.1038/oby.2005.130

[4] Sims EA. Are there persons who are obese, but metabolically healthy? Metabolism 2001;50:1499–1504. doi: 10.1053/meta.2001.27213 11735101 10.1053/meta.2001.27213

[5] Arnlov J, Sundstrom J, Ingelsson E, Lind L. Impact of BMI and the metabolic syndrome on the risk of diabetes in middle-aged men. Diabetes Care 2011;34:61–65. doi: 10.2337/dc10-0955 20852030 10.2337/dc10-0955 PMC3005442

[6] Epel ES, Lin J, Wilhelm FH, Wolkowitz OM, Cawthon R, Adler NE, et al Cell aging in relation to stress arousal and cardiovascular disease risk factors. Psychoneuroendocrinology 2006;31:277–287. doi: 10.1016/j.psyneuen.2005.08.011 16298085 10.1016/j.psyneuen.2005.08.011

[7] Cawthon RM, Smith KR, O’Brien E, Sivatchenko A, Kerber RA. Association between telomere length in blood and mortality in people aged 60 years or older. Lancet 2003;361:393–351. doi: 10.1016/S0140-6736(03)12384-7 12573379 10.1016/S0140-6736(03)12384-7

[8] Blackburn EH. Telomere states and cell fates. Nature 2000;408:53–56. doi: 10.1038/35040500 11081503 10.1038/35040500

[9] Hastie ND, Dempster M, Dunlop MG, Thompson AM, Green DK, Allshire RC. Telomere reduction in human colorectal carcinoma and with ageing. Nature 1990;346:866–868. doi: 10.1038/346866a0 2392154 10.1038/346866a0

[10] Iwama H, Ohyashiki K, Ohyashiki JH, Hayashi S, Yahata N, Ando K, et al Telomeric length and telomerase activity vary with age in peripheral blood cells obtained from normal individuals. Hum Genet 1998;102:397–402. 9600234 10.1007/s004390050711

[11] Satoh H, Hiyama K, Takeda M, Awaya Y, Watanabe K, Ihara Y, et al Telomere shortening in peripheral blood cells was related with aging but not with white blood cell count. Jpn J Hum Genet 1996;41:413–417. doi: 10.1007/BF01876332 9088112 10.1007/BF01876332

[12] Harley CB, Futcher AB, Greider CW. Telomeres shorten during ageing of human fibroblasts. Nature 1990;345:458–460. doi: 10.1038/345458a0 2342578 10.1038/345458a0

[13] Zglinicki V. Oxidative stress shortens telomeres. Trends Biochem Sci 2002;27:339–344. 12114022 10.1016/s0968-0004(02)02110-2

[14] Armanios M. Syndromes of telomere shortening. Annu Rev Genom Hum Genet 2009;10:45–61. 10.1146/annurev-genom-082908-150046 PMC2818564 19405848

[15] Gardner JP, Li S, Srinivasan SR, Chen W, Kimura M, Lu X, et al Rise in insulin resistance is associated with escalated telomere attrition. Circulation 2005;111:2171–2173. doi: 10.1161/01.CIR.0000163550.70487.0B 15851602 10.1161/01.CIR.0000163550.70487.0B

[16] Lee M, Martin H, Firpo MA, Demerath EW. Inverse association between adiposity and telomere length: The Fels Longitudinal Study. Am J Hum Biol 2011;23:100–106. doi: 10.1002/ajhb.21109 21080476 10.1002/ajhb.21109 PMC3245638

[17] Satoh M, Ishikawa Y, Takahashi Y, Itoh T, Minami Y, Nakamura M. Association between oxidative DNA damage and telomere shortening in circulating endothelial progenitor cells obtained from metabolic syndrome patients with coronary artery disease. Atherosclerosis 2008;198:347–353. doi: 10.1016/j.atherosclerosis.2007.09.040 17983621 10.1016/j.atherosclerosis.2007.09.040

[18] Cassidy A, De Vivo I, Liu Y, Han J, Prescott J, Hunter D, et al Associations between diet, lifestyle factors, and telomere length in Women. Am J Clin Nutr 2010;91:1273–1280. doi: 10.3945/ajcn.2009.28947 20219960 10.3945/ajcn.2009.28947 PMC2854902

[19] Nordfjall K, Eliasson M, Stegmayr B, Melander O, Nilsson P & Roos G. Telomere length is associated with obesity parameters but with a gender difference. Obesity (Silver Spring) 2008;16:2682–2689. 18820651 10.1038/oby.2008.413

[20] Vilariño J, Gónzalez C, Granceli H, Damiano M, Frechtel G, Costa Gil J, et al Increased prevalence of type 2 diabetes and obesity in central Argentina (1997–2010): A systematic multistage population based study. Venado Tuerto 2 study (VT 2). Revista ALAD 2015;4:140–147.

[21] Alberti KGMM, Eckel RH, Grundy SM, Zimmet P, Cleeman JI, Donato KA, et al Harmonizing the Metabolic Syndrome: A Joint Interim Statement of the International Diabetes Federation Task Force on Epidemiology and Prevention; National Heart, Lung, and Blood Institute; American Heart Association; World Heart Federation; International Atherosclerosis Society; and International Association for the Study of Obesity. Circulation 2009;120:1640–1645. doi: 10.1161/CIRCULATIONAHA.109.192644 19805654 10.1161/CIRCULATIONAHA.109.192644

[22] Grundy SM, Brewer HB Jr, Cleeman JI, Smith SC Jr, Lenfant C. Definition of metabolic syndrome: Report of the National Heart, Lung, and Blood Institute/American Heart Association conference on scientific issues related to definition. Circulation 2004;109:433–4317. doi: 10.1161/01.CIR.0000111245.75752.C6 14744958 10.1161/01.CIR.0000111245.75752.C6

[23] Cherkas LF, Hunkin JL, Kato BS, Richards JB, Gardner JP, Surdulescu GL, Kimura M, Lu X, Spector TD, Aviv A. The Association Between Physical Activity in Leisure Time and Leukocyte Telomere Length. Arch Intern Med 2008;168:154–158. doi: 10.1001/archinternmed.2007.39 18227361 10.1001/archinternmed.2007.39

[24] Verde Z, Reinoso-Barbero L, Chicharro L, Garatachea N, Resano P, Sánchez-Hernández I, Rodríguez González-Moro JM, Bandrés F, Santiago C, Gómez-Gallego F. Effects of cigarette smoking and nicotine metabolite ratio on leukocyte telomere length. Environmental Research 2015;140:488–494. doi: 10.1016/j.envres.2015.05.008 25996625 10.1016/j.envres.2015.05.008

[25] Ridker PM. Clinical application of C-reactive protein for cardiovascular disease detection and prevention. Circulation 2003;107:363–369. 12551853 10.1161/01.cir.0000053730.47739.3c

[26] O´Callaghan NJ, Fenech M. A quantitative PCR method for measuring absolute telomere length. Biological Procedures Online 2011;13:3 doi: 10.1186/1480-9222-13-3 21369534 10.1186/1480-9222-13-3 PMC3047434

[27] Julieta Panero J, O’Callaghan NJ, Fenech M, Slavutsky I. Absolute qPCR for measuring telomere length in bone marrow samples of plasma cell disorders. Mol Biotechnol 2014;57(2):155–159. 10.1007/s12033-014-9811-8 25311116

[28] Karelis AD, St-Pierre DH, Conus F, Rabasa-Lhoret R, Poehlman ET. Metabolic and body composition factors in subgroups of obesity: what do we know? J Clin Endocrinol Metab 2004;89(6):2569–2575. doi: 10.1210/jc.2004-0165 15181025 10.1210/jc.2004-0165

[29] Shaharyar S, Roberson LL, Jamal O, Younus A, Blaha MJ, Ali SS. Obesity and metabolic phenotypes (metabolically healthy and unhealthy variants) are significantly associated with prevalence of elevated C-reactive protein and hepatic steatosis in a large healthy Brazilian population. J Obes 2015;2015:178526 doi: 10.1155/2015/178526 25838943 10.1155/2015/178526 PMC4369939

[30] Gao X, Zhang W, Wang Y, Pedram P, Cahill F, Zhai G, Randell E, Gulliver W, Sun G. Serum metabolic biomarkers distinguish metabolically healthy peripherally obese from unhealthy centrally obese individuals. Nutrition and Metabolism 2016;13:33–43. doi: 10.1186/s12986-016-0095-9 27175209 10.1186/s12986-016-0095-9 PMC4865032

[31] Hamer M, Stamatakis E. Metabolically healthy obesity and risk of all-cause and cardiovascular disease mortality. J Clin Endocrinol Metab 2012;97:2482–2488. doi: 10.1210/jc.2011-3475 22508708 10.1210/jc.2011-3475 PMC3387408

[32] Aung K, Lorenzo C, Hinojosa MA, Haffner SM. Risk of developing diabetes and cardiovascular disease in metabolically unhealthy normal-weight and metabolically healthy obese individuals. J Clin Endocrinol Metab 2014;99:462–468. doi: 10.1210/jc.2013-2832 24257907 10.1210/jc.2013-2832 PMC3913817

[33] Kramer CK, Zinman B, Retnakaran R. Are metabolically healthy overweight and obesity benign conditions?: A systematic review and meta-analysis. Ann Intern Med 2013;159:758–769. doi: 10.7326/0003-4819-159-11-201312030-00008 24297192 10.7326/0003-4819-159-11-201312030-00008

[34] Satoh M, Ishikawa Y, Takahashi Y, Itoh T, Minami T, Nakamura M. Association between oxidative DNA damage and telomere shortening in circulating endothelial progenitor cells obtained from metabolic syndrome patients with coronary artery disease. Atherosclerosis 2008;198:347–353. doi: 10.1016/j.atherosclerosis.2007.09.040 17983621 10.1016/j.atherosclerosis.2007.09.040

[35] Révész D, Milaneschi Y, Verhoeven J, Penninx B. Telomere length as a marker of cellular ageing is associated with prevalence and progression of metabolic syndrome. J Clin Endocrinol Metab 2014;99:1–10. 25188715 10.1210/jc.2014-1851

[36] Njajou OT, Cawthon RM, Blackburn EH, Harris TB, Li R, Sanders JL, et al Shorter telomeres are associated with obesity and weight gain in the elderly. Int J Obes (Lond) 2012;36:1176–1179. 22005719 10.1038/ijo.2011.196 PMC3408817

[37] Rehkopf DH, Needham BL, Lin J, Blackburn EH, Zota AR, Wojcicki JM, et al Leukocyte Telomere Length in Relation to 17 Biomarkers of Cardiovascular Disease Risk: A Cross-Sectional Study of US Adults. PLoS Med 2016;13(11):e1002188 doi: 10.1371/journal.pmed.1002188 27898678 10.1371/journal.pmed.1002188 PMC5127504

[38] Valdes AM, Andrew T, Gardner JP, Kimura M, Oelsner E, Cherkas LF, et al Obesity, cigarette smoking, and telomere length in women. Lancet 2005;366:662–664. doi: 10.1016/S0140-6736(05)66630-5 16112303 10.1016/S0140-6736(05)66630-5

[39] Brouilette SW, Moore JS, McMahon AD, Thompson JR, Ford I, Shepherd J, et al Telomere length, risk of coronary heart disease, and statin treatment in the West of Scotland Primary Prevention Study: a nested case-control study. Lancet 2007;369:107–114. doi: 10.1016/S0140-6736(07)60071-3 17223473 10.1016/S0140-6736(07)60071-3

[40] Bekaert S, De Meyer T, Rietzschel ER, De Buyzere ML, De Bacquer D, Langlois M, et al Telomere length and cardiovascular risk factors in a middle-aged population free of overt cardiovascular disease. Aging Cell 2007;6:639–647. doi: 10.1111/j.1474-9726.2007.00321.x 17874998 10.1111/j.1474-9726.2007.00321.x

[41] Serra V, Grune T, Sitte N, Saretzki G, von Zglinicki T. Telomere length as a marker of oxidative stress in primary human fibroblast cultures. Ann NY Acad Sci 2000;908:327–330. 10911978 10.1111/j.1749-6632.2000.tb06666.x

[42] Révész D, Milaneschi Y, Verhoeven JE, Lin J, Penninx BW. Longitudinal Associations Between Metabolic Syndrome Components andTelomere Shortening. J Clin Endocrinol Metab 2015;100:3050–3059. doi: 10.1210/JC.2015-1995 26133009 10.1210/JC.2015-1995

[43] falta bibliografia Navarro E, Funtikova AN, Fíto M, Schröder H. Can metabolically healthy obesity be explained by diet, genetics, and inflammation? Mol Nutr Food Res 2015;59:75–93. doi: 10.1002/mnfr.201400521 25418549 10.1002/mnfr.201400521

[44] Martin-Ruiz CM, Baird D, Roger L, Boukamp P, Krunic D, Cawthon R, et al Reproducibility of Telomere Length Assessment-An International Collaborative Study. Int J Epidemiol 2015;44(5):1749–1754. doi: 10.1093/ije/dyv171 26403809 10.1093/ije/dyv171 PMC6312091

[45] Mathew H, Farr O, Mantzoros C. Metabolic health and weight: Understanding metabolically unhealthy normal weight or metabolically healthy obese patients. Metab Clin Exp 2016;65:73–80. 10.1016/j.metabol.2015.10.019 PMC4750380 26683798

[46] Sanders JL, Newman AB. Telomere length in epidemiology: a biomarker of aging, age-related disease, both, or neither? Epidemiologic reviews 2013;35(1):112–131. 23302541 10.1093/epirev/mxs008 PMC4707879

